# Healing X-ray scattering images

**DOI:** 10.1107/S2052252517006212

**Published:** 2017-05-24

**Authors:** Jiliang Liu, Julien Lhermitte, Ye Tian, Zheng Zhang, Dantong Yu, Kevin G. Yager

**Affiliations:** a Center for Functional Nanomaterials, Brookhaven National Laboratory, Upton, New York 11973, USA; b Computational Science Initiative, Brookhaven National Laboratory, Upton, New York 11973, USA; c New Jersey Institute of Technology, Newark, New Jersey 07102, USA

**Keywords:** X-ray scattering, SAXS, image healing, inpainting, data completion

## Abstract

X-ray scattering images contain numerous gaps and defects arising from detector limitations and experimental configuration. We present a method to heal X-ray scattering images, filling gaps in the data and removing defects in a physically meaningful manner. Unlike generic inpainting methods, this method is closely tuned to the expected structure of reciprocal-space data. In particular, we exploit statistical tests and symmetry analysis to identify the structure of an image; we then copy, average and interpolate measured data into gaps in a way that respects the identified structure and symmetry. Importantly, the underlying analysis methods provide useful characterization of structures present in the image, including the identification of diffuse *versus* sharp features, anisotropy and symmetry. The presented method leverages known characteristics of reciprocal space, enabling physically reasonable reconstruction even with large image gaps. The method will correspondingly fail for images that violate these underlying assumptions. The method assumes point symmetry and is thus applicable to small-angle X-ray scattering (SAXS) data, but only to a subset of wide-angle data. Our method succeeds in filling gaps and healing defects in experimental images, including extending data beyond the original detector borders.

## Abstract 2

A new method is presented for healing X-ray scattering images, allowing gaps in images to be filled and extending the image beyond the borders of the original detector exposure. This method exploits symmetry analysis to reconstruct the sample structure and thereby fill-in unmeasured regions of reciprocal space based on this automatically determined symmetry model.

## 1. Introduction   

X-ray scattering is a powerful technique for quantifying structural order in materials. Small-angle X-ray scattering (SAXS) enables probing of nanoscale structural order, while wide-angle X-ray scattering (WAXS) can probe molecular and atomic order. These techniques have seen broad uptake for studying a wide variety of materials (Williams *et al.*, 1999 ▸), including hard matter such as alloys, ceramics and composites (Fratzl, 2003 ▸); soft matter (Hexemer & Müller-Buschbaum, 2015 ▸), including polymers (Higgins & Stein, 1978 ▸; Chu & Hsiao, 2001 ▸; Smilgies *et al.*, 2002 ▸) and interfacial ordering phenomena (Cristofolini, 2014 ▸); nanomaterials (Dubcek, 2005 ▸) such as block-copolymers (Müller-Buschbaum, 2003 ▸; Müller-Buschbaum, 2016 ▸; Majewski & Yager, 2016 ▸), nanoparticles (Ingham, 2015 ▸; Li *et al.*, 2016 ▸) and nanoparticle superlattices (Yager *et al.*, 2014 ▸; Senesi & Lee, 2015 ▸); and biological materials (Blanchet & Svergun, 2013 ▸; Jacques & Trewhella, 2010 ▸; Yang, 2014 ▸; Vestergaard & Sayers, 2014 ▸), including plant (Liu, Kim *et al.*, 2016 ▸) and human tissues (Liu, Constantino *et al.*, 2016 ▸).

In a modern synchrotron X-ray scattering experiment, data are collected on a two-dimensional (area) detector. Beamlines are increasingly using photon-counting area detectors, which are fabricated by merging together many pixel-array modules to provide tiled coverage of a given solid angle. An experimental X-ray scattering image inevitably contains a host of defects in the area image: bad pixels (either detector defects or ‘zingers’ during the exposure), the beamstop, high-intensity streaks (*e.g.* from slit scattering), detector inter-module gaps and shadowing from the experimental geometry (sample chamber, exit window *etc*.). Collectively, these defects are treated by masking the experimental image, and thereby discarding portions of the data that are erroneous or untrustworthy. These masked regions are – manifestly – gaps in the data; ideally these gaps would instead be filled with physically correct information.

In this paper, we describe an algorithm to fill gaps in X-ray scattering datasets, thereby ‘healing’ the image defects. At first glance, this would seem to be a highly ill-advised operation. The gaps in the data represent segments of reciprocal space that were not measured; thus, nothing can be unambiguously said about the data one would have obtained if those regions had been experimentally measured. Any image correction will thus either introduce substantial artifacts, or will simply reflect the assumptions inherent to the healing algorithm. However, there are in fact a number of reasons why healing of scattering images can be extremely beneficial – provided the correction is done in a way that respects the physics of scattering.

Firstly, we note that image healing can be useful as part of data visualization. The exclusion of known defects and artifacts from X-ray scattering images allows the experimenter to instead focus on the portions of the data that are meaningful. While masking covers untrustworthy data, filling gaps is preferable in the sense that unphysical regions of zero intensity are avoided. Moreover, image healing can be applied in an ‘extension’ or ‘continuation’ mode, where the structure of the image outside of the original image regions is inferred (*e.g.* one can attempt to reconstruct the complete 360° pattern from a single quadrant). Such a visualization can help a human experimenter to identify the structure and symmetry of the scattering pattern more easily.

Secondly, image completion allows X-ray scattering detector data to be used in a wider range of software. Software written specifically for X-ray scattering, diffraction and crystallography is typically ‘mask aware,’ with explicit handling for regions of data to be included/excluded from analysis (Yang, 2013 ▸; Jiang, 2015 ▸; Hammersley, 2016 ▸). Complete scattering images can, however, additionally be transformed, processed and analyzed using the enormous range of existing image analysis algorithms, software libraries and graphic tools. For instance, most existing implementations of correlation analysis and convolution algorithms are not mask aware; yet these tools can yield important insights about sample structure (Wochner *et al.*, 2009 ▸; Altarelli *et al.*, 2010 ▸; Lhermitte *et al.*, 2017 ▸). It is also worth noting that computational performance is likely to be higher for implementations that can ignore masking issues.

Thirdly, image healing can be extremely beneficial for automated analysis. Modern synchrotrons and X-ray free electron lasers (XFELs) have unprecedented brightness and are yielding data at previously unimaginable rates (Weckert, 2015 ▸; Schlichting, 2015 ▸). It is no longer practical for human scientists to analyze these enormous data volumes manually; instead, automated analysis is increasingly being pursued. Machine-learning methods have made enormous progress in recent years in a wide variety of domains, including text recognition, machine translation, automated transcription and image identification (Lecun *et al.*, 1998 ▸; Krizhevsky *et al.*, 2012 ▸). Recently, these kinds of tools have been applied to solve scientific data challenges, including in the analysis of scattering images (Yoon *et al.*, 2011 ▸; Giannakis *et al.*, 2012 ▸; Schwander *et al.*, 2012 ▸). In our ongoing work on the use of machine vision and deep learning methods to categorize X-ray scattering images automatically (Huang *et al.*, 2014 ▸; Kiapour *et al.*, 2014 ▸; Wang, Guan *et al.*, 2016 ▸; Wang, Yager *et al.*, 2016 ▸), we have found that image masking can be a substantial hindrance. From the point of view of image recognition, the mask represents an extremely strong feature, exhibiting sharp edges with high intensity variation. These features can easily dominate the analysis (refer to Fig. S1 in the supporting information for an example; where in a PCA analysis, all of the components exhibit the mask features, since these features are strong and invariant). Image healing is thus a useful preprocessing step, removing an artifact that would otherwise confuse machine-learning methods. Moreover, image extension can be useful to bring all training and testing images into a uniform space. That is, X-ray scattering data are measured in a highly heterogeneous way (sampling somewhat different windows of reciprocal space, depending on beamline setup); image extension can map this heterogeneous data into a uniform representation. Overall, our preliminary results indicate that preprocessing of X-ray scattering images is an extremely useful tool for vastly improving the performance of machine-learning methods.

Finally, as shall be seen throughout this manuscript, the implementation of an image-healing algorithm tuned to X-ray scattering data inherently extracts intermediate results that are of value in the analysis of such data. A robust image-healing workflow can be thought of instead as an image analysis workflow, where the ultimate healed image is ignored and the intermediate analysis results are used instead. In this sense, image correction is an excellent test case to develop improved analysis methods and pipelines.

Image healing is a well studied problem, especially as it applies to photographs (*e.g.* removing dust and scratches), scanned documents and photo retouching. In order to fill small gaps in images, a variety of inpainting algorithms have been developed (Bertalmio *et al.*, 2003 ▸; Bugeau & Bertalmio, 2009 ▸). The simplest algorithms involve interpolation using linear or polynomial fits. While such methods can fill small gaps in smooth and continuous data, they fail outside of these simple cases. This approach can be improved by taking inspiration from transport equations, thereby modeling ‘flow’ of image intensity into gaps (Casaca *et al.*, 2014 ▸). Structural inpainting extrapolates the geometric structure of the image (Bertalmio *et al.*, 2000 ▸), while textural inpainting replicates repeating patterns into gaps. These two approaches can be combined for improved quality (Bertalmio *et al.*, 2003 ▸; Sangeetha *et al.*, 2011 ▸). Another class of approaches focuses on identifying best matches between patches adjacent to gaps and patches elsewhere in the image (Criminisi *et al.*, 2003 ▸, 2004 ▸). While these algorithms can achieve impressive results when applied to photographs, they generally perform poorly for scientific datasets, and X-ray scattering images in particular. Firstly, the gaps in scattering images can be quite large (compared to what simple inpainting methods can accommodate without artifacts). Moreover, most conventional approaches do not correctly handle the (experimentally common) case of an important feature being truncated by a mask edge, or entirely lost behind a masked region. For instance, a Gaussian peak bisected by a gap will not be correctly reconstructed by interpolation (which will simply connect the shoulders of the peak). More advanced algorithms may improve the visual quality of the reconstruction, but they generally will not do so in a way that actually matches the nature of the experimental data.

Here, we develop a ‘physics-aware’ image-healing algorithm. By exploiting fundamental properties of reciprocal space (such as continuity and symmetry) and typical experimental features (such as peaks and rings), our algorithm reconstructs in a way that conforms to the known physics of scattering experiments. We demonstrate robust statistical methods for extracting a signature of overall image features (*e.g.* diffuse *versus* localized) and for determining the symmetry of features. We again emphasize that filling gaps in a detector image is a formally ill-posed problem, without a well defined single solution. Nevertheless, by using a healing algorithm closely tied to the nature of scattering data, we are able to both inpaint and extend scattering images in a manner that preserves – and indeed highlights – the real structural features of the data.

## 2. Methods and results   

### 2.1. Approach   

**Figure 1:**
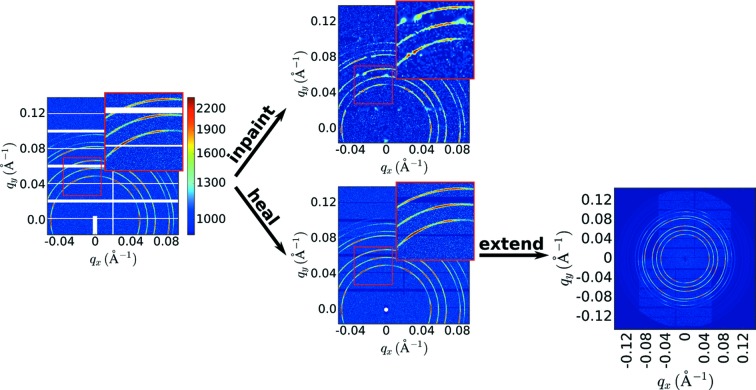
X-ray scattering area detector images include numerous regions of missing or untrustworthy data, which must be masked (left image, white regions). A simple inpainting algorithm (from the *scikit-image* library) fills gaps using interpolation (upper image). This yields poor results because the structure inherent to the scattering data is ignored. We present an algorithm that exploits symmetry analysis to heal images in a ‘physics-aware’ mode (bottom image). By exploiting the symmetry of scattering data, the filled regions connect correctly with the measured data. This algorithm can moreover be used to ‘extend’ the image well beyond the borders of the original detector (bottom right). This highlights the overall structure and symmetry of the original data.

X-ray scattering area detector images may have numerous regions of missing or untrustworthy data, which are masked for further analysis (white regions in Fig. 1 ▸, left). A straightforward method to inpainting is to interpolate across gaps in the data. However, such methods yield poor results when applied to masked scattering images (Fig. 1 ▸, top). The gaps in scattering images can be quite large and naive interpolation cannot recover important features that have been truncated or entirely lost. Moreover, such methods ignore the structure inherent to the scattering image. For instance, scattering images have a well defined origin, with a great number of image features being coupled to this origin (*e.g.* rings arc around the origin). We also note that the computational performance of traditional algorithms on scattering data is quite poor. Because of the large size of scattering images (*e.g.* megapixels) and the wide gaps of masked regions, inpainting algorithms execute rather slowly. (In fact, for the example in Fig. 1 ▸, the overall image quality is reduced because the image needed to be downsampled in order for the algorithm to complete in a reasonable time.) Although more advanced algorithms can improve the inpainting quality, by ignoring the structure of scattering data, they inevitably yield a reconstruction that is not physically meaningful.

Here, we present a ‘physics-aware’ image-healing algorithm, which exploits the properties of scattering datasets to reconstruct in a physically sound manner. For instance, rings of scattering intensity can be analyzed to estimate their symmetry and this knowledge can be used to copy data from a measured part of the image into a masked region in a way that respects the measured symmetry. Our method (Fig. 1 ▸) out-performs existing algorithms when applied to X-ray scattering data, as it is able to fill image gaps in a way that maintains image continuity and – more importantly – respects the structure of the sample. Because the reconstruction is based on an estimated structure for reciprocal space, the image can moreover be extended even beyond the borders of the original detector image.

Because X-ray scattering data can be highly complex, with scattering contributions from multiple sources, we developed a pipeline (supporting Fig. S2) that first analyzes the overall structure in the image, in order to estimate what kinds of features are present (sharp peaks, diffuse background *etc*.). Subsequently, these specific features are analyzed in turn, fitting each component in order to yield estimates for these components into the masked regions. Over the course of this analysis, our pipeline incidentally computes a variety of extremely useful metrics regarding the structure in the image. Even if the healed image itself is not desired, these analysis results provide a useful fingerprint of the scattering data.

In this work, we assume images exhibit point symmetry, 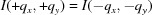. Reciprocal space is centrosymmetric and thus in the small-angle limit the detector image (which is a cut through the three-dimensional reciprocal space) will exhibit point symmetry. Many wide-angle scattering patterns will also exhibit point symmetry; *e.g.* for powder-like samples, or where the material’s symmetry axes are appropriately aligned with the experimental geometry. In general, however, wide-angle scattering images need not exhibit point symmetry, because of the curvature of the Ewald sphere (Breiby *et al.*, 2008 ▸; Baker *et al.*, 2010 ▸).

We implemented our workflow in the Python programming language, taking advantage of existing Python libraries for efficient numerical computations [*numpy* (Oliphant, 2007 ▸)], fitting (*scipy* and *lmfit*), image analysis [*scikit-image* (van der Walt *et al.*, 2014 ▸)] and plotting [*matplotlib* (Hunter, 2007 ▸)]. To test our method, we use both simulated and experimental data. The simulated data (Yager *et al.*, 2017 ▸) allow us to systematically vary image features in order to test hypotheses and optimize our workflow; the experimental data provide a rigorous test of the validity of our approach even on the messy, inconsistent and heterogeneous data encountered at a modern synchrotron beamline. Simulated images were generated using an *ad hoc* approach; that is, summing together computed contributions for various features typically seen in scattering images (isotropic rings, diffuse halos, peaks organized along rings or forming a well defined lattice *etc*.).

The input to our software is the detector image, the corresponding mask and the beam center position. The workflow outputs the image with masked regions filled-in and (optionally) an extended version of the image going beyond the original detector boundary. The input mask can be minimal (*e.g*. only the intermodule gaps), or can include untrustworthy parts of the image (bad pixels, parasitic streaks *etc*.). In this sense, our algorithm acts as both a gap-filling method and a defect-removal method.

### 2.2. Structural identification   

**Figure 2:**
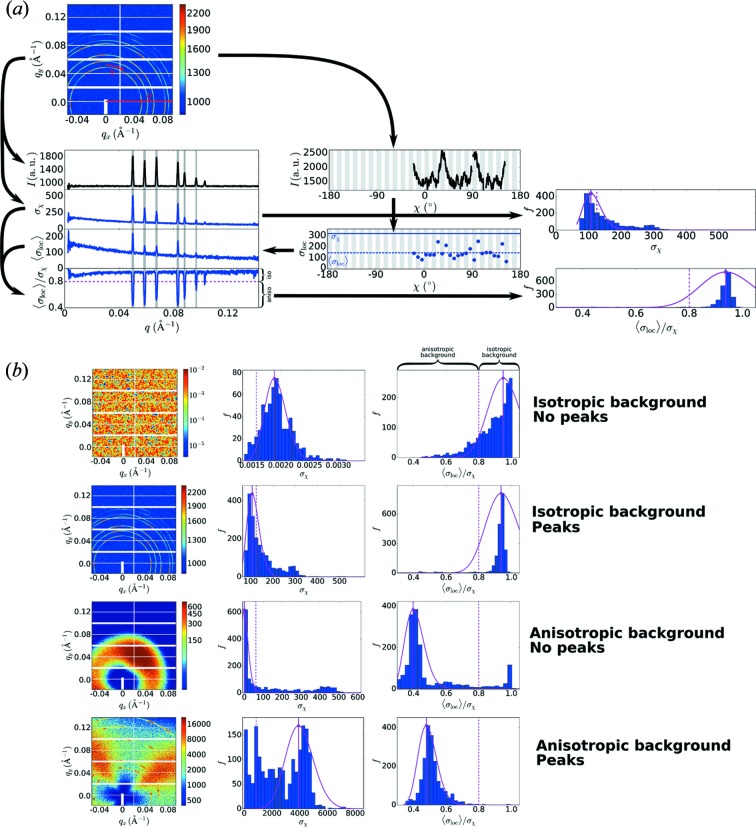
(*a*) Method to quantify structural features. For each *q* value, the intensity along the angular direction, 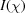, can be analyzed. The standard deviation of this entire curve (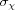) can be compared with the average of the ‘local’ standard deviations along this curve (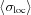). The ratio of these quantities provides a measure of whether 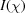 is structured: one expects 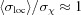 for an isotropic curve and 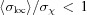 for an anisotropic curve. The histograms (right) of 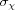 and 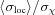 (accumulated across all *q*) provide a signature of the overall structure in the image. (*b*) Method to classify images. When the 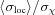 histogram is peaked near 1.0, the background can be inferred to be isotropic. When the 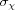 histogram is skewed (relative to the 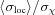 histogram), one can infer the presence of sharp anisotropic peaks in the data.

The starting point for healing an X-ray scattering image is to identify the types of structures that appear in the image. We differentiate between diffuse and broad scattering patterns (which we can label as the ‘background’) and more sharp and local patterns, including most peaks and rings (which we can analogously refer to as ‘foreground’ features). Both diffuse and sharp features can be either isotropic or anisotropic; *i.e.* uniform along the azimuthal angular direction (χ), or angularly structured. We thus classify each input image into one of four possible categories (Fig. 2 ▸ *b*): purely isotropic patterns, images with isotropic background but anisotropic peaks overlaid, anisotropic patterns with purely diffuse features, or complex images containing both a structured background and structured sharp features.

Fig. 2 ▸(*a*) shows the analysis steps used to quantify image structures. For each *q* value in the image, we extract the intensity along the azimuthal angular direction, 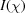. From each of these curves, we compute the overall standard deviation of the curve, 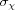, as well as a series of local standard deviations, 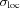 (we empirically select a binning width of 7.5°). We then average these local standard deviations to obtain 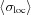. Intuitively, if a curve is uniform (isotropic), then the local and overall standard deviations will be similar. Any discrepancy between these values suggests that the curve is locally smoother than it is overall; 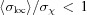 implies it is structured (anisotropic at that *q*). We empirically select a threshold value of 0.8 to differentiate between regions along *q* where the scattering pattern is isotropic *versus* anisotropic. Thus, regions where 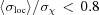 can be identified as anisotropic and flagged for subsequent symmetry analysis. By using a ratio of variances (rather than absolute variances), we normalize against the enormous variability observed among different detector images. This procedure also normalizes against the non-uniform sampling across the image; different *q* regions have both different intensities and different numbers of available pixels.

The calculation of these standard deviations can be pooled to yield a signature of the overall structure in the image (Fig. 2 ▸ *b*). In particular, we generate histograms of both 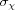 and 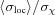. For automated analysis of these histograms, we fit them to a Poisson distribution: 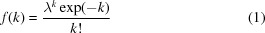
where *f* is frequency, *k* is the bin index for the histogram variable (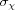 or 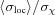) and λ is a fit parameter, which we use to quantify the center of the distribution (the Poisson λ provides a better estimate than the mean because the distributions can be highly skewed). We empirically select a histogram with 30 bins, spanning from the minimum to the maximum of the distribution. Fitting using the bin index inherently normalizes the distributions to a common (albeit arbitrary) scale; we convert the computed λ values back to the corresponding histogram variable (and denote them as 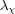 and 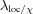).

When 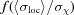 is peaked near 1.0, we can infer that the majority of the image is angularly unstructured. Thus, when 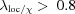, we conclude that the background is isotropic. If the background is isotropic, we identify the existence of peaks by inspecting 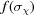. If there are no anisotropic peaks, then 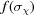 resembles a balanced normal distribution and 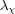 is close to the center of the distribution span. On the other hand, if anisotropic peaks are present in the data, this will add larger values of 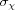 to the distribution, which will create a ‘long tail’ in the distribution. This will move 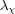 towards the low end of the distribution (relative to the overall span of the distribution). We consider the relative position of the distribution’s center, 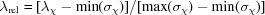; our empirically developed criterion is that when 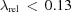, we conclude that anisotropic peaks are superimposed on the isotropic background.

If the background has been identified as anisotropic, the interpretation of 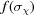 is different. Owing to the anisotropy over large *q*-spans, 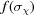 will be highly skewed, with a substantial ‘long tail,’ and a correspondingly small 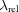. We thus identify purely diffuse scattering patterns as when 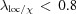 and 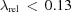. Conversely, the appearance of sharp, anisotropic peaks superimposed on such data will increase the histogram bins at large 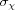 and thereby tend to shift the center of the distribution to larger values (*i.e.* 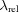 will increase). Thus, we can identify complex scattering patterns (with anisotropic diffuse and localized features) as when 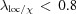 and 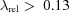.

Overall, these simple statistical metrics provide a robust way to estimate the overall character of the scattering pattern (supporting Fig. S3). These metrics are intrinsically useful as a way to assess a scattering image automatically (to identify anisotropy or the appearance of peaks); we also exploit the metrics in this work to categorize images into the appropriate branch of the healing workflow (supporting Fig. S2).

### 2.3. Healing isotropic data   

The simplest data to consider are those that are essentially isotropic across the entire measured *q*-range. In such a case, the one-dimensional circularly averaged curve (computed by excluding masked pixels) yields a robust estimate of the scattering intensity throughout the entire image. This case by itself is not particularly useful, since the two-dimensional image does not contain any information beyond that captured in the one-dimensional curve. Nevertheless, this operation is a useful tool as part of our complete workflow. In particular, image regions found to be purely isotropic, or that cannot be healed with any other method, can be filled with the one-dimensional curve.

### 2.4. Healing ordered patterns   

A common type of X-ray scattering image is that where the background is smooth and isotropic, but where structural features (peaks, rings *etc*.) are not isotropic. Since our method assumes point symmetry, we can first fill image regions assuming twofold symmetry. That is, we can copy data from 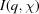 to any gap located at 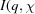 + 180°). This will, of course, not fill all the image gaps. The underlying physics of X-ray scattering causes the majority of structured, anisotropic patterns to exhibit some form of well defined symmetry. In particular, distinct peaks or textured rings arise from scattering from well defined local order (a nanoscale packing motif for SAXS, or a molecular/atomic unit cell for WAXS). The symmetry of this local order is reproduced in the resulting scattering pattern. Even in cases where a scattering ring appears nearly isotropic, the seemingly random fluctuations in the ring are in fact angularly correlated, as they arise from a large population of randomly oriented grains, each exhibiting a well defined unit cell (Lehmkühler *et al.*, 2014 ▸; Ingham, 2014 ▸; Yager & Majewski, 2014 ▸). We exploit this well known characteristic of scattering patterns to fill image gaps.

**Figure 3:**
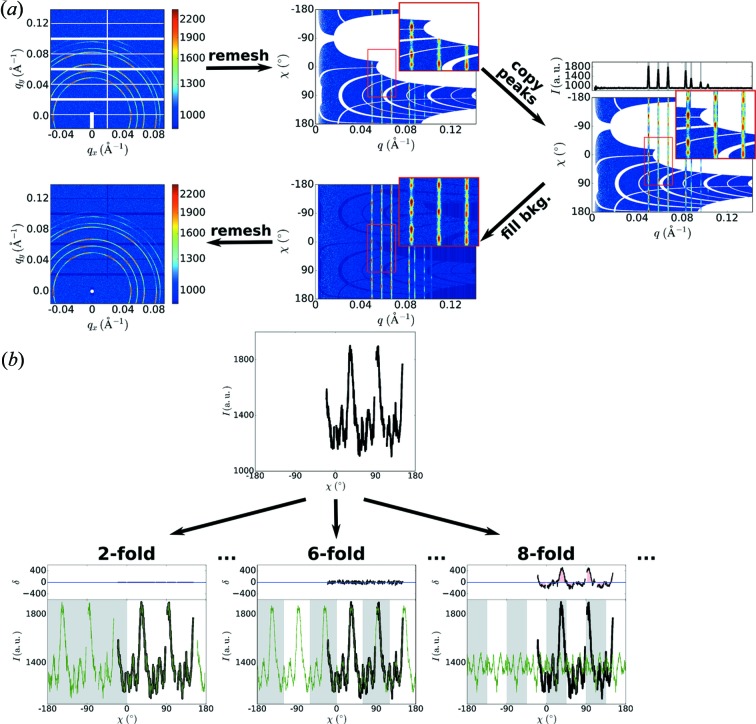
(*a*) Anisotropic peaks are healed using symmetry analysis. The 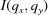 image is remeshed into a 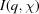 representation. Each peak is analyzed in turn to determine its symmetry. Based on the calculated symmetry, the experimental data for this region are copied repeatedly into gaps (with shifts along χ consistent with the determined symmetry). Any remaining gaps in the isotropic background are filled using the one-dimensional curve (circular average). (*b*) The symmetry of an incomplete 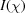 curve is assessed by calculating the residuals between the experimental data and various model curves (3 examples are shown). The model curves are generated by copying and averaging the curve with a shift determined by the symmetry.

As shown in Fig. 3 ▸, we simplify this analysis by remeshing the data from the usual 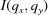 image into an 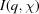 representation. Using the previously described statistical metrics (*cf.* Fig. 2 ▸ *a*), we identify the *q*-ranges where anisotropic peaks are present (supporting Fig. S4). For each peak, we measure the angular symmetry. A variety of methods can be used to assess this symmetry, including a Fourier or spherical harmonic decomposition, or angular correlation methods (Altarelli *et al.*, 2010 ▸; Kurta *et al.*, 2013 ▸; Lehmkühler *et al.*, 2014 ▸; Mendez *et al.*, 2016 ▸). Here, we use a brute-force comparison method, since it is robust, involves very few assumptions and is easily generalizable to assessing other data features. The method (Fig. 3 ▸ *b*) consists of taking the experimental data (black) and comparing them with a variety of models (green) constructed by shifting and averaging the experimental data following different symmetry assumptions. Since we assume point symmetry, we only evaluate even symmetries (2, 4, 6, 8, 10, …). The error between the experimental and candidate *n*-fold model (residuals shown above each comparison) provides a measure of whether the given model is valid. We note that the error will always be smaller for lower symmetry (especially since, *e.g.*, any sixfold symmetric pattern is of course twofold symmetric), whereas we wish to identify the highest symmetry that is consistent with the data. As such, we apply an additional bias to favor cases where there is more overlap in generating the model. In particular, we select the smallest δ defined as: 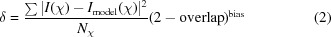
where 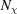 is the number of experimental points [in 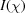]. The ‘overlap’ parameter is the relative amount of overlapped experimental data used to generate the model (*i.e.* the number of points within an *n*-fold span that were averaged, divided by the width of the span). This parameter takes on a value between 0 and 1, with 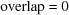 occurring when the *n*-fold copy does not result in any averaging of different parts of the curve (data from one span are copied into gaps in the other spans), while 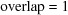 occurs when averaging occurs over the entire span (no gaps). The 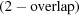 term thus biases towards higher symmetries (which involve more overlap copying). The ‘
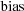
’ exponent controls the relative weighting of this effect; we select 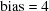 empirically. This regularization term can be fine-tuned for specific datasets. For instance, extremely noisy data will increase the residuals contribution, in which case a larger bias may be necessary in order to correctly identify the highest valid symmetry.

Once the symmetry of a given *q*-range is determined, it can be healed by simply copying the two-dimensional data in 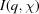 following this computed symmetry for shifting and averaging. As always in our algorithm, we favor experimental data wherever possible. In other words, the symmetry-averaged data are only used to fill masked gaps. Any gaps remaining after this operation is complete are, as always, filled with the one-dimensional circularly averaged curve. The data can then be remeshed back into an 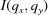 image. As can be seen, this relatively simple sequence of operations succeeds in filling the image gaps in a visually smooth and physically correct manner.

### 2.5. Healing diffuse patterns   

**Figure 4:**
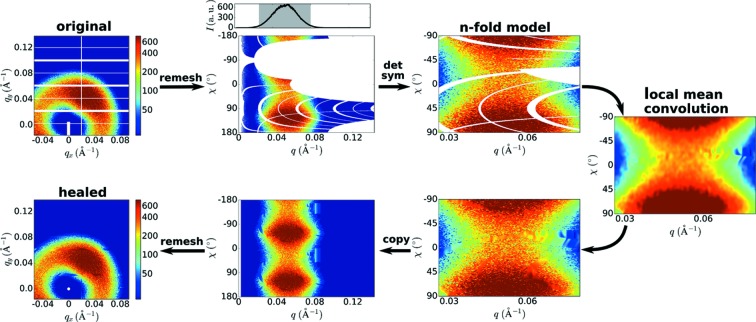
Anisotropic diffuse scattering is healed by combining symmetry analysis with interpolation. The 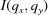 image is remeshed into a 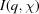 representation. Anisotropic regions are analyzed in turn to determine symmetry; experimental data are averaged into an *n*-fold model. Remaining gaps are filled using interpolation. The filled symmetric model is then copied repeatedly (according to the computed symmetry) to fill the image.

Poorly ordered materials that nevertheless exhibit some kind of preferred packing motif give rise to broad and diffuse rings/halos of scattering intensity. For instance, amorphous polymers will exhibit a WAXS halo, nanoparticle packing will give rise to a SAXS ring and very small crystalline domains will generate very broad diffraction peaks. Orientation of semi-ordered materials (due to shear, stretching, field alignment *etc*.) leads to diffuse scattering that is angularly structured. Our analysis pipeline (*cf.* supporting Fig. S2) first identifies whether such diffuse anisotropic features are present, and if so, then applies an inpainting method tuned to these kinds of structures. Diffuse anisotropic scattering patterns can again be healed using symmetry analysis (Fig. 4 ▸), with the added complication that the breadth of diffuse scattering increases the likelihood of gaps remaining even after symmetry averaging.

In healing diffuse scattering patterns, we again first identify *q*-regions exhibiting anisotropy, using the previously described statistical measure. The symmetry of these regions is computed as described earlier: the experimental data are compared with candidate models for even symmetries. By copying and averaging the 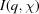 data according to the computed symmetry, we build an *n*-fold model for the data. This model fills regions using available experimental data, averaging where data from multiple parts of the original image are available. Because diffuse scattering is, by definition, spread over a very wide *q*-space, it is likely that gaps remain even after this averaging operation. Given the breadth of the features relative to the gaps, relatively simple and generic interpolate can be used to fill the remaining gaps. In Fig. 4 ▸, we use an iterative local mean convolution filter, which progressively extends the image into gaps, while also smoothing the image. More sophisticated interpolation (*e.g.* two-dimensional polynomial), intensity diffusion, or inpainting methods could be used at this stage, at a cost in execution time.

The filled *n*-fold model can then be copied throughout the image (filling, as always, the isotropic regions with the circular average). This operation yields a completely healed image that respects both the symmetry of the diffuse scattering and the local continuity of the image. We note that although using a simple interpolation method can give rise to artifacts, the scale of these errors is small compared to the overall scattering.

### 2.6. Healing complex patterns   

The most general case in X-ray scattering is a complex pattern exhibiting structured local features (peaks, textured rings *etc*.) and anisotropic diffuse scattering. The symmetry and orientation of these patterns need not coincide. For instance, a structured background can arise due to experimental geometry (sample cell window shadowing *etc*.) unrelated to sample alignment. Structural scattering from distinct coexisting phases exhibiting different symmetry and alignment behavior is also possible.

**Figure 5:**
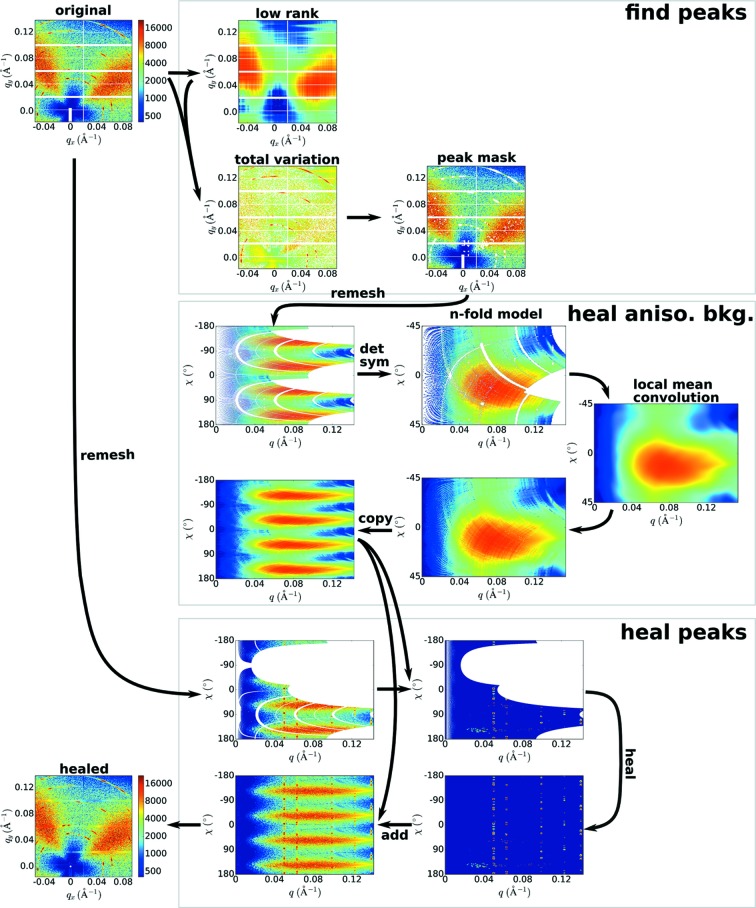
Complex scattering patterns are healed by first separating the scattering into ‘background’ and ‘sharp peak’ components. These two components are healed as demonstrated in previous figures. The image is separated into components by assessing the local variation in the image, in order to identify localized regions of high variance, from which a ‘peak mask’ is constructed.

Our strategy in this case is to separate the experimental scattering intensity into two components (sharp and diffuse) and heal these components separately using the previously described methods (Fig. 5 ▸). In this case, we cannot simply identify the *q*-regions that exhibit sharp peaks, but must instead construct a two-dimensional peak mask that localizes peaks in both *q* and χ. To do this, we first generate an estimate of the background scattering. We use singular value decomposition (SVD), which conceptually represents the input image as a linear combination of basis terms (Golub & Reinsch, 1970 ▸). While the full input image can be reconstructed by combining all the singular values, we can intentionally reconstruct a low-fidelity image using a selected subset of the singular values. By using only the low-rank components (we select only the first two), the reconstruction is intentionally low resolution, reproducing the broad and diffuse features while omitting sharp and local structures. While this provides an estimate of the background scattering, the low-rank matrix contains artifacts arising from the decomposition. In principle, one can subtract this background estimate from the original data and identify peaks as regions of significant intensity variation. However, because noise scales with intensity (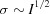), regions of the image with substantial diffuse intensity may exhibit large local variance (even after background subtraction). We thus generate a ‘total variation’ map, by dividing the original diffraction pattern by the low-rank image (rather than subtracting); this technique is extremely useful for highlighting sharp local intensity variations (Vese & Osher, 2003 ▸). The peak mask is then generated by thresholding this variation image.

Once the peak mask is generated, the background and foreground components can be healed as previously described. The background is healed using a mask that is the union of the original mask and the peak mask. Anisotropic background regions are identified and an *n*-fold symmetry model computed. This model is filled *via* interpolation and copied to fill the background component. A peak-only image is then generated by subtracting this background component from the original image. The peak component is healed using symmetry analysis to copy and average the experimental peak intensity into image gaps. Finally, the two components are added together and remeshed back into a 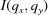 image.

### 2.7. Image extension   

Because the described image-healing method analyzes image structure, it can be used to reconstruct even outside the original image boundaries (*cf.* Fig. 1 ▸). Throughout the workflow, images are healed in an 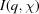 map, which allows features (peaks, diffuse scattering) to be healed across the full χ range. One can thus remesh to 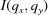 over an extended window to reconstruct the full 360° of each scattering arc. Moreover, we can also attempt to fill in the lingering gaps at low-*q* and high-*q* by fitting the available data to reasonable models (supporting Fig. S5). It is important to note that this aggressive extension into regions outside of that directly measured experimentally is inherently error prone and model dependent. Thus, data reconstructed in this manner should not generally be used as the basis for fitting to extract physical parameters. Any such analysis would be highly dependent on the assumptions that have been built into the correction pipeline. Nevertheless, generating these extended images is useful to emphasize the overall structure of the scattering pattern visually and as an input to analysis methods that cannot tolerate any masking effects, and where model-dependent artifacts are preferable to artifacts resulting from image discontinuities.

## 3. Discussion   

Our image correction method is closely aligned with the structures and features typically seen in X-ray scattering data. While this reduces the generality of the method, it greatly improves the quality of the healing. Our method is ‘physics-aware’ in the sense that the known physics of the X-ray scattering process, the traditional experimental geometry and the typical experimentally observed features, have all been encoded into the assumptions and processing steps of the method. The method is implemented as a workflow of modules, each of which is useful on its own when tackling the analysis of X-ray scattering data (supporting Fig. S2). The method first involves the high-level analysis of the image, identifying anisotropy and local *versus* diffuse structures; this classification is then used to decide what sequence of healing operations is required. Where necessary, the image is split into diffuse (background) and sharp (*e.g.* peaks) components. Sharp peaks are assumed to lie along rings of constant *q* and exhibit a well defined even angular symmetry. Once symmetry is assessed, healed amounts to copying (and, where possible, averaging) existing data into gaps according to the symmetry. Anisotropic diffuse scattering can be similarly healed, with added low-pass filtering and interpolation to fill small gaps. Overall, this relatively simple workflow allows a wide variety of experimentally interesting images to be healed.

**Figure 6:**
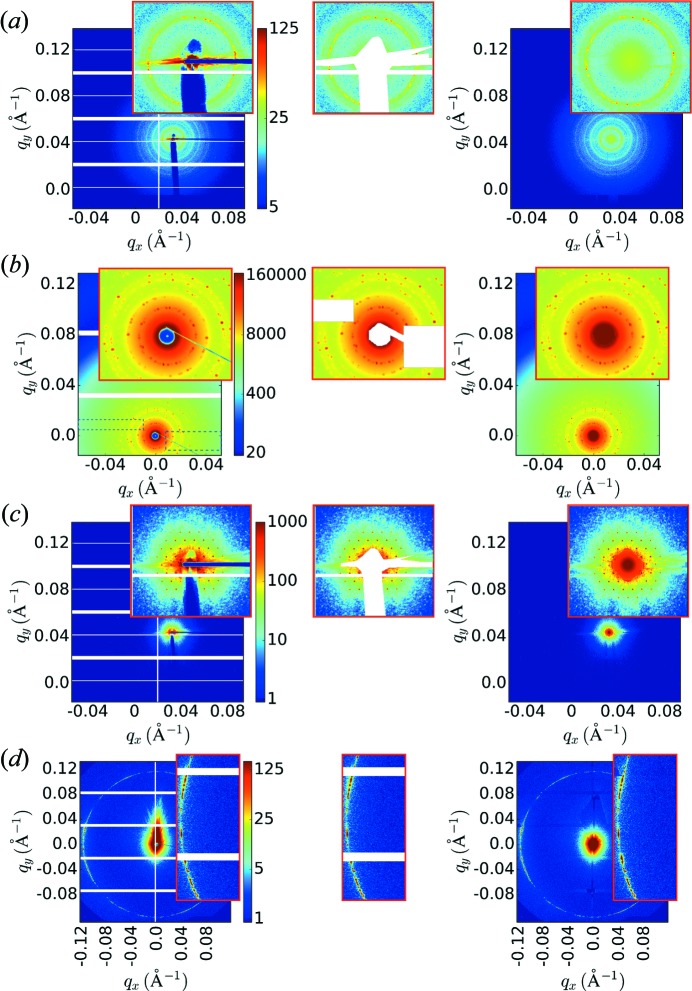
Examples of image healing applied to experimental data (left, original data; center, masking; right, healed image). (*a*), (*b*) Nanoparticle superlattices self-assembled using DNA nanostructures. In (*b*), valid areas of the image (dashed boxes) are intentionally added to the mask (and thereby healed), to demonstrate the capability of the method. (*c*) Hexagonal arrangement of cylindrical nanopores etched into silicon. (*d*) A liquid-crystalline small-molecule forming a weakly aligned poly grain phase. Both missing data regions and corrupted data (*e.g.* streaks at small angle) can be effectively healed.

Fig. 6 ▸ shows example experimental X-ray scattering data healed using this method (including nanoparticle superlattices self-assembled using DNA origami (Tian *et al.*, 2016 ▸), a hexagonal array of nanopores in silicon fabrication using electron-beam lithography and a liquid-crystalline phase (Yager & Majewski, 2014 ▸). As can be seen, the healing is both visually convincing and physically meaningful (additional examples shown in supporting Fig. S6). As a further test of the method, we can intentionally remove valid portions of the data and compare these masked regions to the healed image. Fig. 6 ▸ b (and supporting Fig. S6*a*) shows an example (dashed boxes denote regions intentionally masked), where the reconstruction successfully fills the excluded regions.

This image healing can be used both to fill unavoidable gaps (intermodule gaps, beamstop *etc*.) and to remove defects. Examples are shown in Fig. 6 ▸(*a*,*c*,*d*) where streaks (arising from parasitic slit scattering or from sample holder sidewalls) are added to the mask and thereby eliminated. Individual erroneous pixels (either damaged detector pixels, or spurious counts) can be added to the mask and similarly healed (supporting Fig. S7). Our method is in fact quite robust to the kinds of noise encountered in X-ray scattering area images (Poisson/shot noise, salt and pepper noise *etc*.). The healed images are easier for a human experimenter to visually assess, as image defects (including gaps) are removed, thereby highlighting the meaningful structure of the data. Moreover, the healed images are ideally suited as inputs to image analysis methods (which may not be able to handle masks) and machine learning methods (where any masking leads to a deterioration in performance).

The image-healing workflow inherently involves a variety of analysis modules that produce useful results. Our method provides statistical measures of the global structure in a scattering image, allowing rapid classification of images (*e.g.* structured *versus* purely diffuse scattering). We also demonstrate local structure identification, identifying peaks in the one-dimensional circularly averaged curve, flagging these peaks as isotropic *versus* anisotropic, or generating a two-dimensional mask of all peaks throughout the image. For identified peaks, we demonstrate the estimation of symmetry. We similarly demonstrate the recognition of anisotropic diffuse features and symmetry assignments. Finally, the fitting of low-*q* and high-*q* regions (in order to extend/complete the image) provides useful information about the functional form of the scattering in these regimes. These analysis results are useful to experimenters when trying to understand their X-ray scattering data. Moreover, taken together they provide a unique and physically grounded signature for any particular scattering pattern. The concatenation of these results is thus an ideal input to statistical machine learning methods, such as support vector machines (SVM). Thus, our pipeline can be viewed as a physics-based feature extractor, pre-processing a scattering image into a concise descriptor that can be used for machine learning.

Our healing workflow is not without limitations. The underlying remeshing makes the method sensitive to an arbitrarily selected χ resolution (supporting Fig. S8). If the χ binning is too coarse, the healed image will exhibit significant artifacts (especially at high *q*); if the binning is too fine, the computation time will be increased unnecessarily. Our method relies on several adjustable parameters, which we tuned to empirically yield reasonable results across a wide range of images. While these provide user-adjustable parameters to modulate the output, automated healing may fail for images very different from our test conditions. A core strength and weakness of our approach is its physics-awareness. By explicitly encoding many assumptions into the algorithm, we leverage our domain knowledge and thereby construct an algorithm that achieves excellent quality healing, far in excess of more general inpainting methods (*cf.* Fig. 1 ▸). However, by being closely tied to particular assumptions (point symmetry, well defined symmetry for rings/peaks*etc.*), our method will fail on any data outside of these bounds.

There are numerous avenues for future improvements of the presented method. The most immediate is to remove the assumption of point symmetry, and thereby allow the method to be applied to WAXS and crystallographic images. In these cases, a more generalized symmetry analysis could be performed, which identifies point symmetry (and *n*-fold symmetry) where it exists, but is also able to identify distorted and non-concentric rings, two-dimensional arrays of peak and the correlated peak positions arising from the intersection of the Ewald sphere with a reciprocal-lattice. In these cases, one can take advantage of known crystallographic symmetry, and the known experimental Ewald curvature, to identify peak patterns. A yet more challenging case is to apply these methods in the context of a grazing-incidence (GI) experiment (GISAXS/GIWAXS), where one must also contend with refraction distortion (Busch *et al.*, 2006 ▸; Breiby *et al.*, 2008 ▸; Lu *et al.*, 2013 ▸). Another possibility is to exploit the image-healing algorithm as a denoising method. In the presented implementation, we favored wherever possible the experimental data, only filling gaps. However, the underlying models that are computed for the background scattering (effectively low-pass filtered) and structural peaks (averaged over all available symmetry repetitions), have low noise compared to the input data. Thus, one could exploit the workflow to generate a low-noise estimate of data, without the increase in peak width that accompanies conventional smoothing approaches.

Finally, we wish to emphasize several cautionary notes. The availability of image correction methods should never be considered a substitute for proper design of experiment and analysis. It is obvious that it is far preferable to avoid the appearance of image gaps in the raw experimental data as much as possible. In this sense, all best practices for data collection should be followed as much as possible. For instance, the obstruction from windows or samples cells should be minimized; the beamstop should be made as small and thin as reasonable; for multi-module detectors featuring image gaps, multiple acquisitions with detector offsets should be used to fill gaps; and detector motion should be used to measure (in a tiling mode) as much of the relevant reciprocal space as possible. While the methods presented here are powerful with respect to visualization and pre-processing, the corrected or ‘healed’ images should never be considered as (nor represented to others as) the actual experimental measurement. The data added to the masked parts of the image are inferred in a way that is highly dependent on the assumptions built into the algorithm. Such reconstructed data can be used as a guide, but should not generally be used for quantitative analysis or in the extraction of physical parameters from the data. Data analysis and fitting pipelines should, wherever possible, operate in a mask-aware mode that ignores empty regions rather than analyzing inpainted data. Importantly, the healed images should be clearly identified as having been heavily modified, and the associated mask (which differentiates regions from the original image *versus* inpainted) should be paired with such images. In this regard, accurate metadata are crucial to the use of this and other forms of complicated image correction. The importance of metadata for scientific datasets is being recognized, with several efforts underway to provide guidelines, software and file formats appropriate for X-ray scattering and diffraction data (Könnecke, 2006 ▸; Könnecke *et al.*, 2015 ▸; Kroon-Batenburg *et al.*, 2017 ▸). Both raw and corrected images should be shared with the community, with metadata clearly differentiating between these data types and capturing the workflow that was used for image correction.

## 4. Conclusion   

Inpainting algorithms have proven useful in a variety of domains to restore incomplete images. X-ray scattering data can include a variety of defects and gaps that are distracting to experimenters and problematic in subsequent analysis. Yet, conventional inpainting algorithms perform very poorly on scattering data, as they do not automatically recognize the kinds of features that appear frequently in the data. We have presented a new physics-aware workflow for correcting and ‘healing’ scattering images, which exploits the known structure of these datasets. In particular, our method differentiates between local and diffuse patterns, and reconstructs into missing parts of the data by assuming the scattering pattern exhibits a recognizable symmetry (isotropic, point symmetry, *n*-fold *etc*.). This method allows us to convincingly heal a wide variety of experimentally observed scattering patterns, including isotropic images, diffuse scattering, the rings or peaks arising from structural order. This image correction method can be useful for data visualization and as a pre-processing step for subsequent analysis. Existing software packages targetted towards two-dimensional X-ray scattering images enable analysis in a mask-aware manner, by ignoring data in the masked regions (Yang, 2013 ▸; Jiang, 2015 ▸; Hammersley, 2016 ▸). However, existing software does not allow estimation of data in missing regions. Our image-healing method can be useful to generate datasets for use in software and with algorithms that are not mask aware. In particular, these healed images are useful regularized inputs for statistical analysis and machine-learning methods.

## Supplementary Material

Supporting figures and information. DOI: 10.1107/S2052252517006212/tj5010sup1.pdf
