# Supplementary material for: Healing X-ray scattering images
Source: IUCrJ. 2017 May 24;4(Pt 4):455–65. doi: 10.1107/S2052252517006212 (PMC5571808; doi:10.1107/S2052252517006212)
Supplement: Supplementary file 1 [file m-04-00455-sup1.pdf]

Supporting Information for: Healing x-ray scattering images

Jiliang Liu,[a] Julien Lhermitte,[a] Ye Tian,[a] Zheng Zhang,[a] Dantong Yu[b,c] and Kevin G. Yager [a]*

[a] *Center for Functional Nanomaterials, Brookhaven National Laboratory, Upton, New York 11973 US*, [b] *Computational Science Initiative, Brookhaven National Laboratory, Upton, New York 11973 US*, and [c] *New Jersey Institute of Technology, Newark, New Jersey 07102 US*. *E-mail: kyager@bnl.gov*

1. Masking

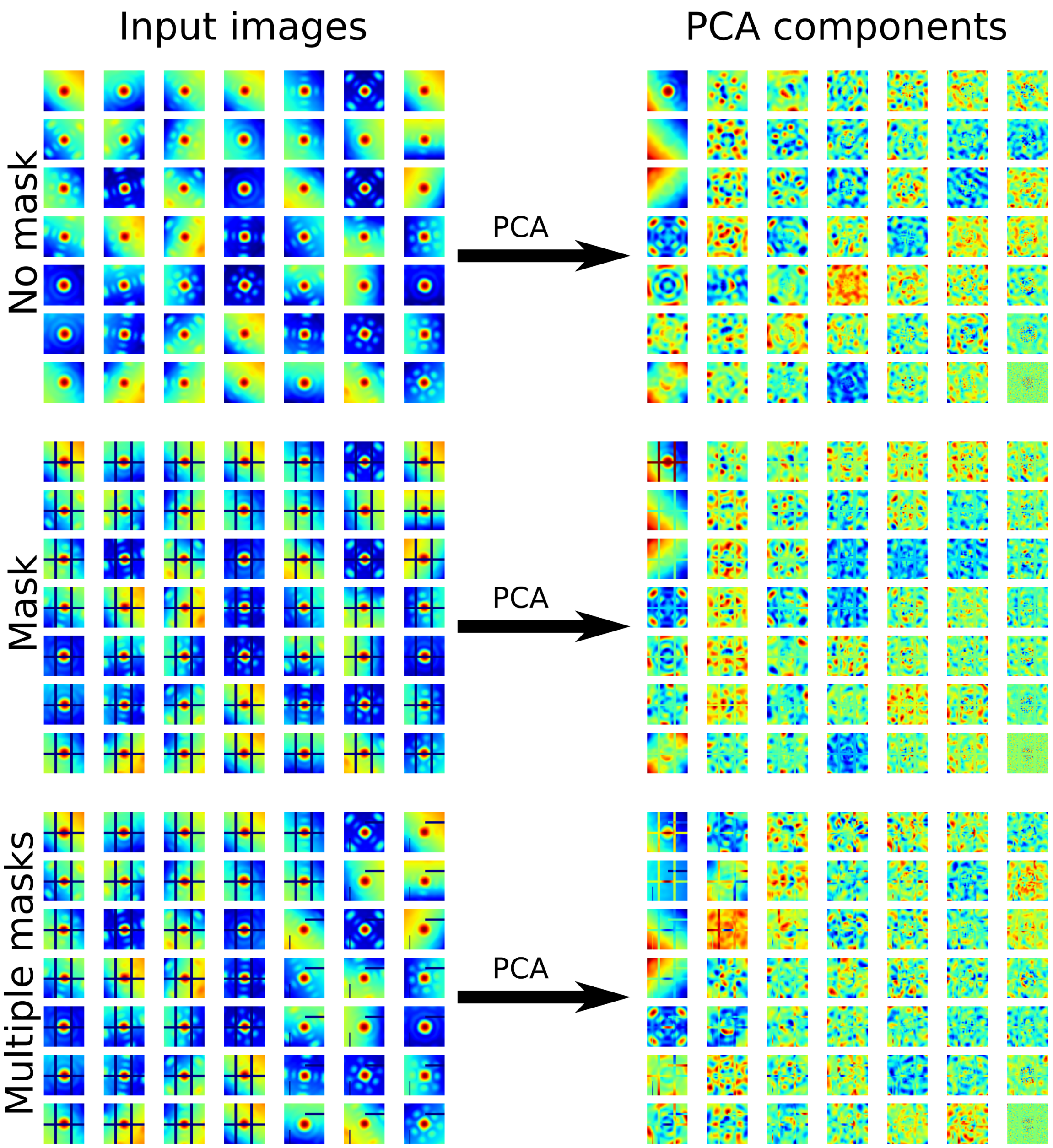

Fig. S1. Masking corrupts subsequent data analysis (including machine learning methods). In this example, principal component analysis (PCA) is used to extract dominant features of the input data. The top row shows how this method extracts leading terms that highlight the symmetry of the input data. The middle row demonstrates that for masked data, every PCA component exhibits the mask. The bottom row demonstrates that when analyzing a mixture of data (which includes different masks), all of the masks present in the data appear in all the PCA components. In other words, the mask corrupts the analysis.

2. Analysis Workflow

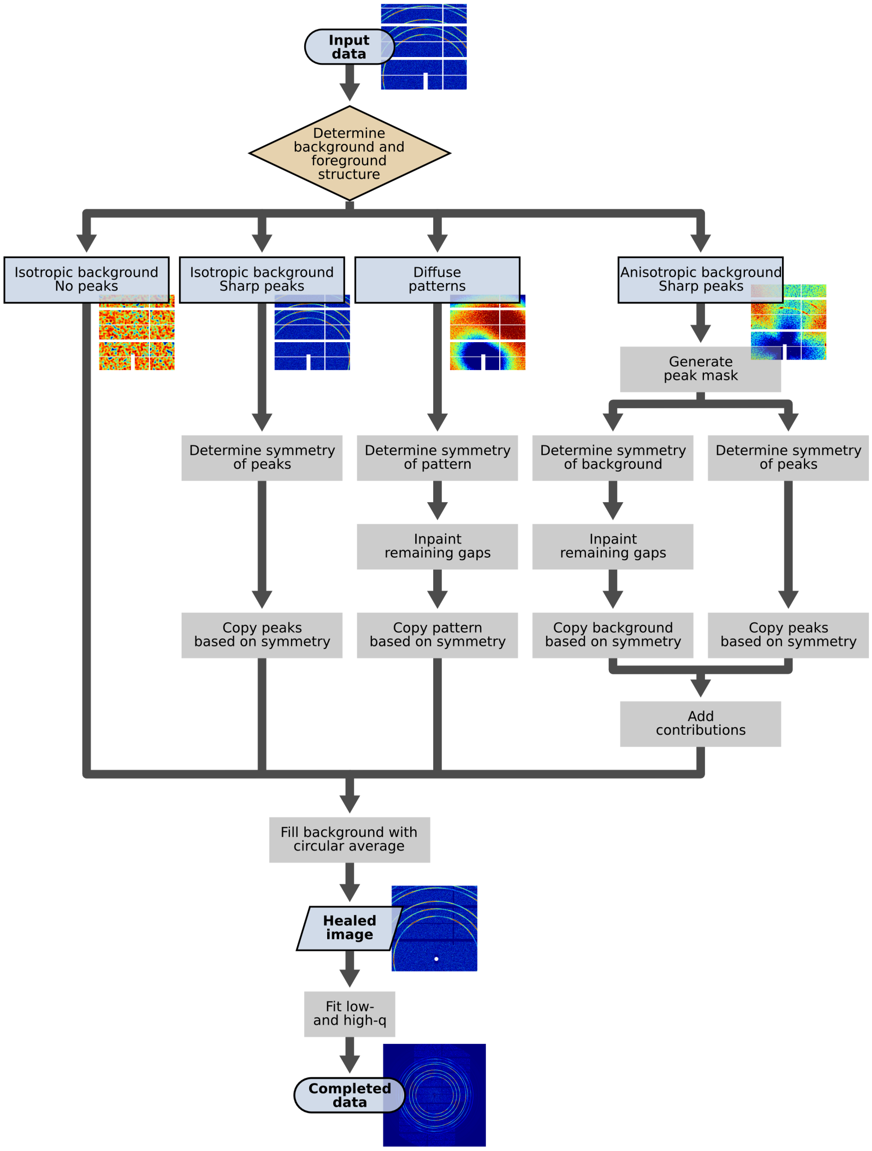

Fig. S2. Analysis workflow for healing an x-ray scattering image. The input image is first analyzed to determine whether the background is structured, and whether there are any sharp 'foreground' features (rings, peaks, etc.). Gaps in the isotropic background can be filled using the 1D circular average curve. Sharp foreground features are analyzed to determine symmetry, and copied throughout the image based on this symmetry. Similarly, purely diffuse anisotropic patterns are be copied based on symmetry. The most complex case is where the background and foreground are both structured (and may have different symmetries). Local structures (peaks) are identified, allowing the background and peaks to be healed separately. As a final step, one can extend the image by also fitting the low-q and high-q regions to reasonable functions.

3. Signatures

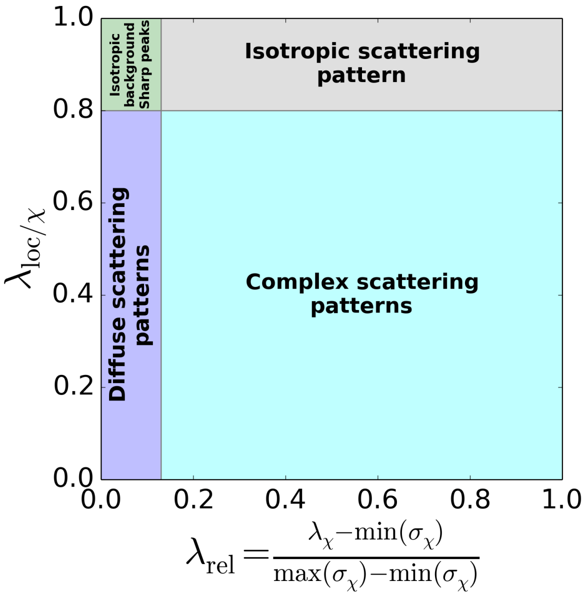

Fig. S3. Signatures used to assess the overall type of a scattering pattern. Two normalized measures of deviation (averaged over the entire image) are computed. $\lambda_{loc/\chi}$ quantifies the average angular homogeneity; $\lambda_{loc/\chi} \approx 1$ indicates a predominantly isotropic image, whereas $\lambda_{loc/\chi} < 1$ implies a significant amount of the image exhibits large angular variance (i.e. the data is anisotropic). λ_{rel} quantifies the distribution of variances. The case $\lambda_{rel} \approx 1 - \lambda_{loc/\chi}$ implies that the distribution of variances is skewed, which indicates that there are sharp local features (peaks) in the data.

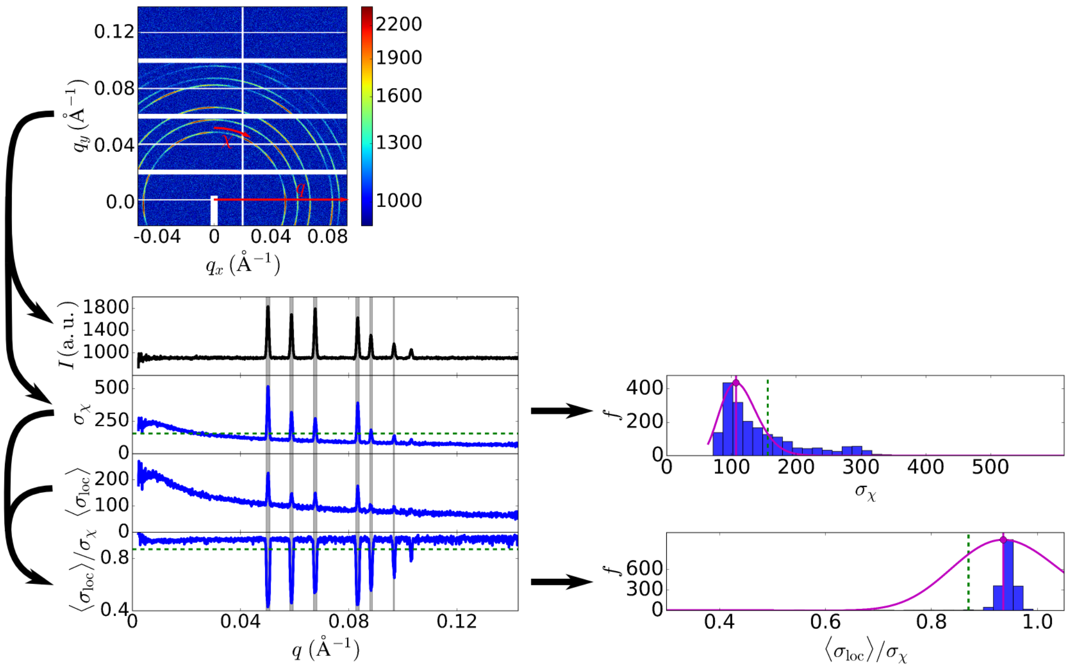

Fig. S4. The existence of structured (anisotropic) peaks in an x-ray scattering image is determined using a statistical test. Two histograms of angular variance are computed: σ_χ (standard deviations along χ) and $\langle\sigma_{\text{loc}}\rangle/\sigma_\chi$ (ratio of average local standard deviation to the overall standard deviation for a curve). Each histogram is fit to a Poisson function, from which we extract a corresponding estimate of central value (λ_χ and $\lambda_{\text{loc}/\chi}$) and standard deviation ($\sim\sqrt{\lambda}$). For each histogram, a threshold is selected based on the Poisson fit (green dashed lines). A q-region is flagged as containing a structured peak when two conditions are met: $\sigma_\chi > \lambda_\chi + a_\chi\sqrt{\lambda_\chi}$ and $\langle\sigma_{\text{loc}}\rangle/\sigma_\chi < \lambda_{\text{loc}/\chi} - a_{\text{loc}/\chi}\sqrt{\lambda_{\text{loc}/\chi}}$, where a_χ and $a_{\text{loc}/\chi}$ are user-adjustable threshold coefficients (which we typically set to 1).

4. Healing modes

The presented image healing method can fill in image regions outside of the original detector borders (Figure S5). Symmetry analysis can be used to copy data across the full χ range (0° to 360°). Such an operation nevertheless leaves a gap at low-q (no data measured behind beamstop) and high-q (no data measured beyond further detector pixel). These regions can be filled by fitting the available data to physically-reasonable functions. For instance, the low-q data can be fit with a Guinier radius-of-gyration

(R_g) model (Hammouda, 2010):

$$I(q) = Ae^{-R_g^2 q^2/3} \tag{1}$$

where A is a normalization constant; or to an Ornstein-Zernike model (Kahlweit *et al.*, 1986; Teubner & Strey, 1987; Nellen *et al.*, 2011):

$$I(q) = \frac{A}{1 + q^2\xi^2} \tag{2}$$

where A is a normalization constant, and ξ is a correlation length; or to a Debye-Bueche random two-phase model (Debye & Bueche, 1949; Debye *et al.*, 1957):

$$I(q) = \frac{A}{(1 + (q\xi)^2)^2} \tag{3}$$

The high-q data can be fit to a Porod fractal law (Ruland, 1971; Koberstein *et al.*, 1980):

$$I(q) = Sq^{-(6-d)} \tag{4}$$

where S is a normalization constant (proportional to surface area), and d is dimensionality. The variable exponent in this equation allows one to fit a variety of data phenomenologically. Fitting the low-q and high-q regions allows the data to be extended to the origin ($q = 0$) and beyond the image boundary. Of course, extending data well beyond the measured data is inherently error-prone and model-dependent. Nevertheless, these extended images are preferable in cases where masking artifacts or image gaps cannot be tolerated.

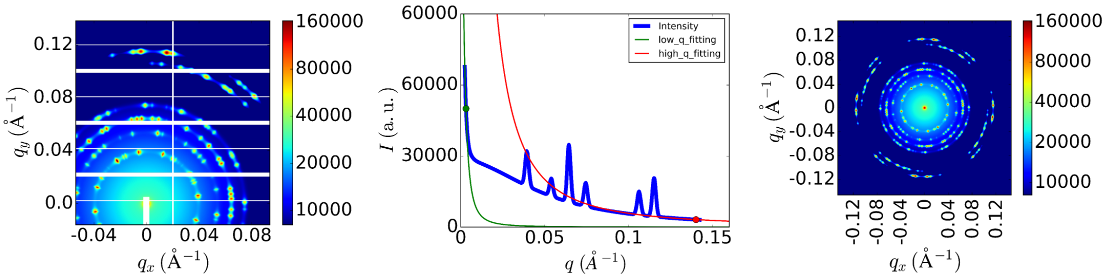

Fig. S5. Image healing can be used to extend an x-ray scattering image beyond the nominal borders of the original detector. The image is extended across all χ using symmetry analysis. Remaining gaps at low-q and high-q are filled by fitting the corresponding q-regions to known functions. In the example shown, the low-q data is fit to an Ornstein-Zernike model, and the high-q to a Porod fractal law.

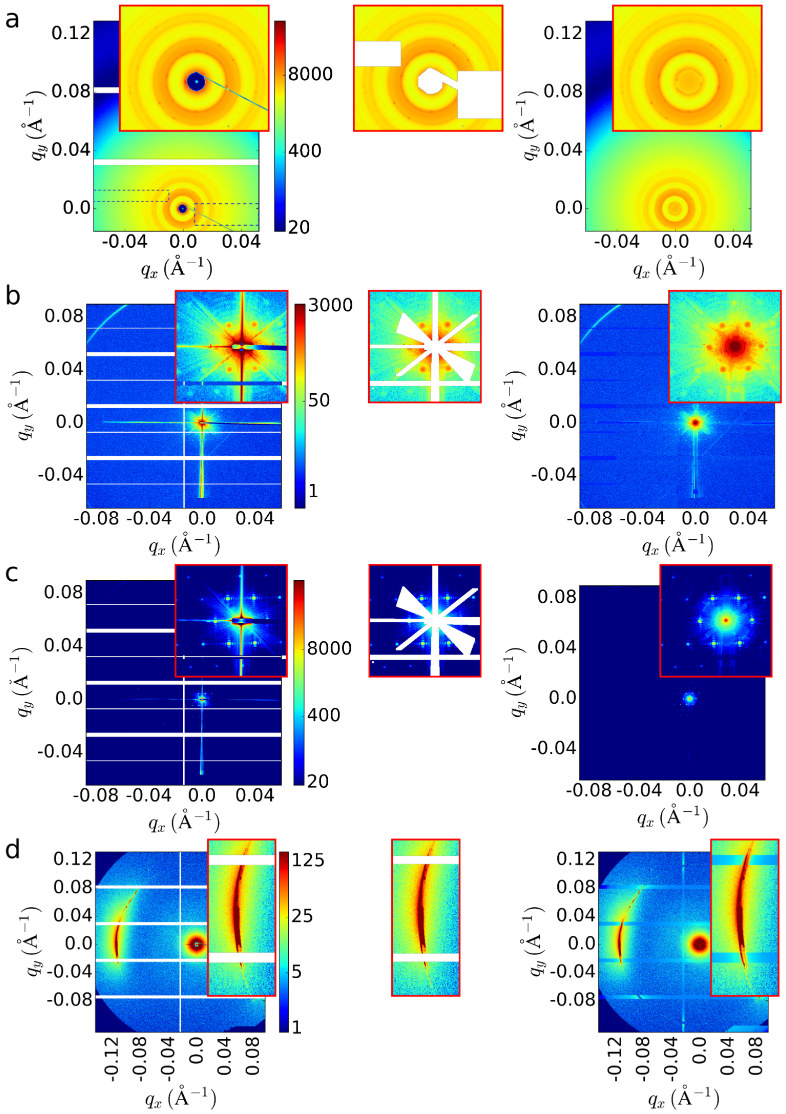

Fig. S6. Examples of image healing applied to experimental data (left, original data; center, masking; right, healed image). (a) Nanoparticle superlattices self-assembled using DNA nanostructures. Valid areas of the image (dashed boxes) are intentionally added to the mask (and thereby healed), to demonstrate the capability of the method. (b, c) Hexagonal arrangement of Au dots (fabricated using electron-beam lithography). (d) A liquid-crystalline small-molecule forming a weakly-aligned poly-grain phase. These examples were selected to highlight certain failure modes. In (b) and (c), the significant amount of masking (and the symmetry of the mask) completely obscures certain features, making a perfect reconstruction impossible. Example (c) also highlights how the method may not identify certain sub-features (e.g. inter-peak fringes). In (d), both the sharp structural peak, and the weaker diffuse halo, are reconstructed. Nevertheless, the intermodule gaps can still be easily seen, since the intensity (and noise) are not perfectly matched along boundaries.

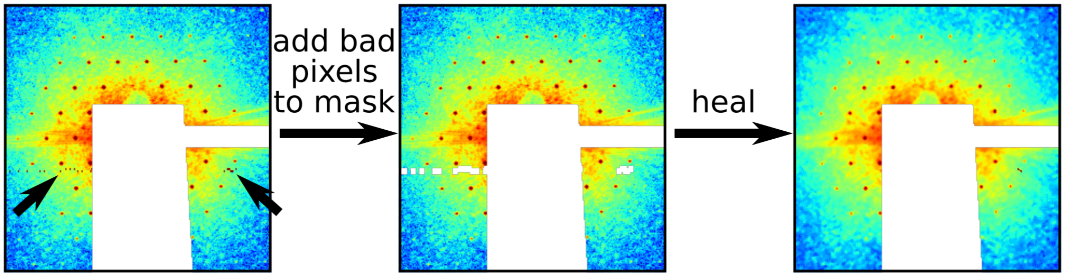

Fig. S7. Image healing can be used to fill erroneous (or 'hot') pixels, which may be intrinsic to a particular detector, or be spurious counts resulting from, e.g., cosmic rays ('zingers'). By adding these pixels to the mask, they are healed and thus eliminated from the image. In the example shown, bad pixels at the boundary between sub-modules in a Dectris Eiger 4M detector (denoted by arrows) are identified, masked, and healed. High-intensity pixels can be automatically flagged by identifying pixels with intensity far greater than their neighbors. We use the criteria $I - m(I * \mathbf{M}) > 0$, where I is the image (intensities), $*\mathbf{M}$ denotes convolution with a median filter, and m is an adjustable threshold parameter (using a sufficiently large m will avoid erroneously flagging experimental intensity variation, while still capturing hot pixels).

5. Binning effects

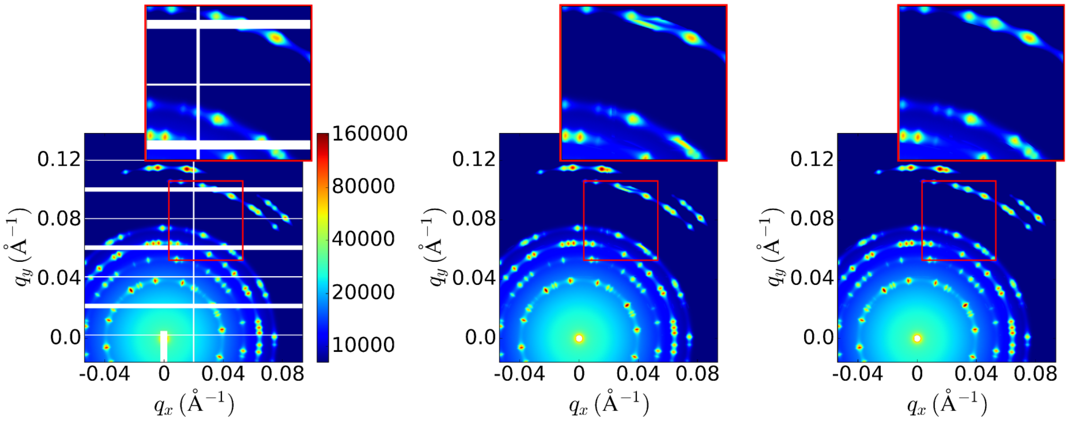

Fig. S8. The transformation from the $I(q_x, q_y)$ image to a $I(q, \chi)$ map involves remeshing with a particular χ resolution. At small-q, this leads to individual experimental pixels being stretched (interpolated) across multiple $I(q, \chi)$ bins, whereas at high-q, this may lead to multiple experimental pixels being combined (averaged) into a single $I(q, \chi)$ bin. While the former is computationally wasteful, the latter represents information loss. This down-sampling (reduction in resolution at high-q) can of course affect the reconstruction quality. In the provided example, the original data (left) is reconstructed using two candidate χ resolutions: 0.5° (center) and 0.1° (right). As can be seen, if the χ resolution is insufficient, the healing algorithm introduces significant artifacts.

Acknowledgements

This research used resources of the Center for Functional Nanomaterials, and the National Synchrotron Light Source II, which are U.S. DOE Office of Science Facilities, operated at Brookhaven National Laboratory under Contract No. DE-SC0012704. Experimental data used in this work was collected at the X9 beamline of NSLS, and the Coherent Hard X-ray (CHX, 11-ID) and Complex Materials Scattering (CMS, 11-BM) beamlines at NSLS-II.

References

Debye, P., Anderson, H. R. & Brumberger, H. (1957). *Journal of Applied Physics*, **28**(6), 679–683.

Debye, P. & Bueche, A. M. (1949). *Journal of Applied Physics*, **20**(6), 518–525.

Hammouda, B. (2010). *Journal of Applied Crystallography*, **43**(4), 716–719.

Kahlweit, M., Strey, R. & Firman, P. (1986). *The Journal of Physical Chemistry*, **90**(4), 671–677.

Koberstein, J. T., Morra, B. & Stein, R. S. (1980). *Journal of Applied Crystallography*, **13**(1), 34–45.

Nellen, U., Dietrich, J., Helden, L., Chodankar, S., Nygrd, K., Friso van der Veen, J. & Bechinger, C. (2011). *Soft Matter*, **7**(11), 5360.

Ruland, W. (1971). *Journal of Applied Crystallography*, **4**(1), 70–73.

Teubner, M. & Strey, R. (1987). *The Journal of Chemical Physics*, **87**(5), 3195–3200.
